# Piezoelectric Energy Harvesting in Internal Fluid Flow

**DOI:** 10.3390/s151026039

**Published:** 2015-10-14

**Authors:** Hyeong Jae Lee, Stewart Sherrit, Luis Phillipe Tosi, Phillip Walkemeyer, Tim Colonius

**Affiliations:** 1 Jet Propulsion Laboratory, California Institute of Technology, Pasadena, CA 91109, USA; E-Mail: Phillip.E.Walkemeyer@jpl.nasa.gov; 2 California Institute of Technology, Pasadena, CA 91109, USA; E-Mails: ltos@caltech.edu (L.P.T.); colonius@caltech.edu (T.C.)

**Keywords:** piezoelectric, flow energy harvesting, fluid-structure interaction, transducer

## Abstract

We consider piezoelectric flow energy harvesting in an internal flow environment with the ultimate goal powering systems such as sensors in deep oil well applications. Fluid motion is coupled to structural vibration via a cantilever beam placed in a converging-diverging flow channel. Two designs were considered for the electromechanical coupling: first; the cantilever itself is a piezoelectric bimorph; second; the cantilever is mounted on a pair of flextensional actuators. We experimentally investigated varying the geometry of the flow passage and the flow rate. Experimental results revealed that the power generated from both designs was similar; producing as much as 20 mW at a flow rate of 20 L/min. The bimorph designs were prone to failure at the extremes of flow rates tested. Finite element analysis (FEA) showed fatigue failure was imminent due to stress concentrations near the bimorph’s clamped region; and that robustness could be improved with a stepped-joint mounting design. A similar FEA model showed the flextensional-based harvester had a resonant frequency of around 375 Hz and an electromechanical coupling of 0.23 between the cantilever and flextensional actuators in a vacuum. These values; along with the power levels demonstrated; are significant steps toward building a system design that can eventually deliver power in the Watts range to devices down within a well.

## 1. Introduction

A variety of devices have been developed to extract energy from the environment through piezoelectric, electromagnetic and thermoelectric energy conversion [1,2,3,4,5,6]. We are interested in energy harvesting to power systems deep in an oil well where ambient pressures of 200 MPa and temperatures higher than 160 °C can occur. Among various transduction mechanisms, vibration-based piezoelectric energy harvesting is attractive for such an application due to the availability of piezoelectric materials with Curie temperatures in the appropriate range as well as their high electromechanical coupling constants. A piezoelectric energy harvester allows many distinct vibration modes to be used to generate electrical power [7], and can be operated with limited strain and wear to promote longevity. 

**Figure 1:**
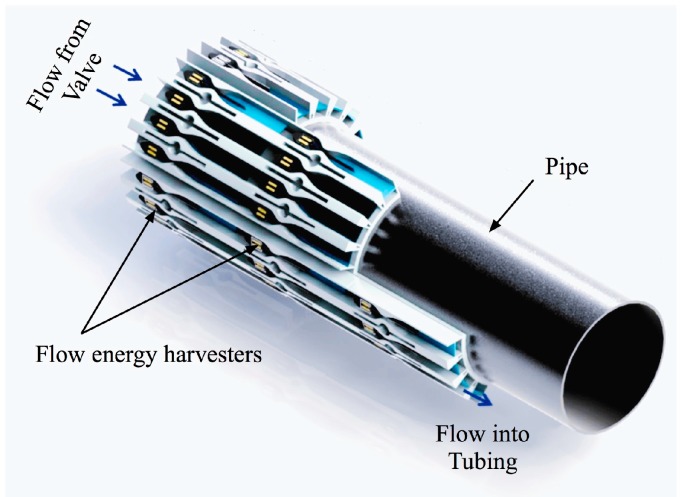
Example of a design concept for a flow energy harvesting system in downhole.

A variety of studies have assessed methods to convert flow energy into vibration. These include vortex shedding [8,9,10,11,12], flapping motions [13], hydraulic pressure [14], and impeller structures [15,16]. The approach we are investigating consists of a cantilever mounted in an internal flow channel where either the cantilever itself is a piezoelectric bimorph, or a non-piezoelectric cantilever drives a transducer [17,18]. The internal flow passage is a spline-shaped nozzle-diffuser. Figure 1 shows the concept design for an array of piezoelectric energy harvester segments that may be combined in series and/or parallel flow paths (and electrically) to generate power levels required by devices down in an oil well. In a standard well with a flow-control valve, the flow enters an annulus between the formation (reservoir rock) and an internal metal pipe (completion tubing), flows through an annular valve, enters the center tubing, and is carried to surface. The illustration makes use of the fluid flow to generate electricity in this annular section.

Clearly, a piezoelectric energy harvester segment with high energy-conversion efficiency from fluid flow to electricity is beneficial, as it reduces both the complexity of system design electronics and the pressure loss in an internal flow environment. Bimorph cantilever-type piezoelectric harvesters are typically used in existing vibration-induced energy harvesting devices [19,20,21]. Since power output is linked to internal stresses generated within the piezoelectric material, the advantage of this type of harvester is its low transverse bending stiffness, which can create large stresses with small amplitude forces relative to other piezoelectric actuators. A maximum output power can be achieved when the harvesting device vibrates at its resonant frequency; thus, a low resonant frequency harvester (<1 kHz) is generally preferred for both robustness to fatigue (less cycles per total lifetime) and ease of coupling to a mechanical system (resonances usually <1 kHz). However, a major drawback is its brittle nature, precipitating short lifetimes due to low fatigue limits. These problems can be mitigated with ruggedized designs, which include the addition of both passive and active mechanisms for limiting the maximum vibration stress/strain levels.

A different approach that improves on the robustness of a bimorph-type piezoelectric harvester is the implementation of a flextensional as the transduction actuator. A flextensional actuator is generally comprised of a multilayer piezoelectric stack inside a metal frame. Since the stack is kept under a compressive load during operation, its design offers a high fatigue limit and a high energy density transducing structure: a lifetime test of piezoelectric stacks revealed no catastrophic failures or degradation after 100 billion cycles [22]; another reported that piezoelectric stacks are capable of producing high electrical power, specifically in the order of 200–300 mW [23]. 

Our flextensional-based flow energy harvester uses a non-piezoelectric cantilever in a converging-diverging flow channel. The cantilever is mounted between two flextensional actuators at its clamped end, with its free end extending downstream of the channel throat. The channel geometry, fluid and flow properties, along with cantilever geometry and material properties dictate the fluid-structure force amplitude and driving frequency at the cantilever’s clamped end. The flextensional resonant frequencies must match those driven by the mounted cantilever, which couples to the fluid flow and provides the forcing function to the actuators. The dimension of the flextensional metal frame governs its resonant frequencies; thus, a frequency-matched flextensional system can be designed and fabricated by controlling the frame’s length and thickness. The integration of flextensional actuators facilitates a design where the piezoelectrics are completely isolated from the working/producing fluids, reducing the effects of corrosion/degradation on the piezoelectric material. 

The emphasis of this work is to design a robust system, such as a highly efficient, piezoelectric harvester that is capable of generating suitable electrical power for sensors and actuators through a range of well production flow rates. This paper presents the preliminary results of experimental and simulation data for various designed, modeled, and prototyped flow energy harvesters.

## 2. Theoretical Background

A cantilever beam with two piezoelectric layers in parallel connection (bimorph), where the two piezoelectric layers have the same polarization directions, has been used to design the initial prototype set of energy harvesting devices. The voltage is generated between the intermediate electrode and the top/bottom electrodes. The constitutive equations describing the behavior of the cantilever type piezoelectric bimorph were first derived by Smits *et al.* [24,25], where the deflection at the free end, δ, and the charge on the electrode, Q, are related to an applied force at the free end, F and an applied voltage over the electrodes, V through a 2 × 2 matrix. The matrix equation under static conditions can be written:

(1) $$\left(\begin{array}{c} \delta \\ Q \end{array}\right)=\left(\begin{array}{cc} \frac{s_{11}^{E}L^{3}}{2wh^{3}} & -\frac{3d_{31}L^{2}}{4h^{2}}\\ -\frac{3d_{31}L^{2}}{4h^{2}} & \frac{2\varepsilon_{33}^{T}wL}{h}(1-\frac{k_{31}^{2}}{4}) \end{array}\right)\left(\begin{array}{c} F\\ V \end{array}\right)$$

where $d_{31}$, $k_{31}$, and $s_{11}^{E}$ are the piezoelectric strain coefficient, electromechanical coupling, and elastic compliance in matrix notation, respectively. $\varepsilon_{33}^{T}$ is the dielectric permittivity. L, w, and h are length, width, and thickness of a piezoelectric plate, respectively.

If an external force F is acting at the bimorph tip (free end), and no voltage is present at the deflected end, (*i.e.*, closed circuit), the generated electrical charge can be expressed as follows:

(2) $$Q_{b}=-\frac{3d_{31}L^{2}}{4h^{2}}F$$

The generated open circuit voltage can be obtained by using the following relation:

(3) $$V_{\text{OC,b}}=\frac{Q}{C_{p}}=\frac{3}{8}(\frac{L}{wh})\frac{d_{31}}{\varepsilon_{33}^{T}}F$$

where $C_{p}$ is the static capacitance of a piezoelectric plate,

(4) $$C_{p}=\frac{\varepsilon_{33}^{T}A}{t}$$

where A is the surface area of a piezoelectric plate, t is the thickness of the composite beam (*t* = 2 h).

From the constitutive equation, Equation (1), the stiffness of bimorph, defined as $k=\frac{F}{\delta }$, under closed circuit condition (V=0) is given by:

(5) $$k=(\frac{s_{11}^{E}L^{3}}{2wh^{3}}{)}^{-1}$$

Since the bimorph is generally composed of several protective layers, the beam stiffness can be expressed as a function of effective Young’s modulus ($Y_{b}$) of composite beam:

(6) $$k_{eq}=\frac{Y_{b}wt^{3}}{4L^{3}}$$

Considering the bimorph in a short-circuit condition as a single degree of freedom spring-mass system, a natural resonant frequency arises as $\omega =\sqrt{\frac{k}{m}}$; thus, the natural frequency of the undamped composite beam ($\omega_{b}$) is given by:

(7) $$\omega_{b}=\sqrt{\frac{Y_{b}wt^{3}}{4m_{eq}L^{3}}}\approx \frac{t}{L^{2}}\sqrt{\frac{Y_{b}}{\rho }}$$

where m_eq_ is an equivalent mass placed at the free end of the cantilever beam and ρ is the density of the composite bimorph.

For a piezoelectric bar operating in a 33 mode, the stress is parallel to the polarization direction, and the generated voltage can be obtained using the piezoelectric constitutive equation:

(8) $$E_{3}=-g_{33}T_{3}+\beta_{33}^{T}D_{3}$$

where $T_{3}$ and $D_{3}$ are the stress on the element in the direction of polarization and dielectric displacement, $E_{3}$ is electric field (=V/t), $\beta_{33}^{T}$ is free dielectric impermeability constant. For the piezoelectric voltage coefficient $g_{33}$, the following equation can be used:

(9) $$g_{33}=\frac{d_{33}}{\varepsilon_{33}^{T}}$$

Under open-circuit condition ($D_{3}=0$), Equation (8) reduces to:

(10) $$|V_{OC,l}|=\frac{d_{33}}{\varepsilon_{33}^{T}}tT_{3}\;\text{=}\;\frac{d_{33}}{C_{p}}F_{3}$$

The generated electrical charge of a longitudinal bar is linearly proportional to the piezoelectric charge coefficient under an applied force, and is expressed as $Q_{l}=d_{33}F_{3}$.

The natural frequency of a longitudinal piezoelectric bar ($\omega_{l}$) is given by:

(11) $$\omega_{l}=\sqrt{\frac{Y_{l}}{\rho }}$$

where $Y_{l}$ is the Young’s modulus of the poled piezoelectric bar, which is the reciprical of $s_{33}^{E}$.

For the case of multilayer piezoelectric stack, where a number of thin alternately poled piezoelectric layers are connected mechanically in series and electrically in parallel, the effective piezoelectric strain coefficient and capacitance is proportional to the number of the piezoelectric layers given by:

(12) $$d_{33}^{*}=nd_{33}$$

(13) $$\varepsilon_{33}^{T*}=\frac{nL\varepsilon_{33,piezo}^{T}}{t_{p}}\approx n^{2}\varepsilon_{33,piezo}^{T}$$

where n is a number of a piezoelectric layers, L and $t_{p}$ are the total length of the piezoelectric stack and the thickness of a single piezoelectric layer, respectively.

**Figure 2:**
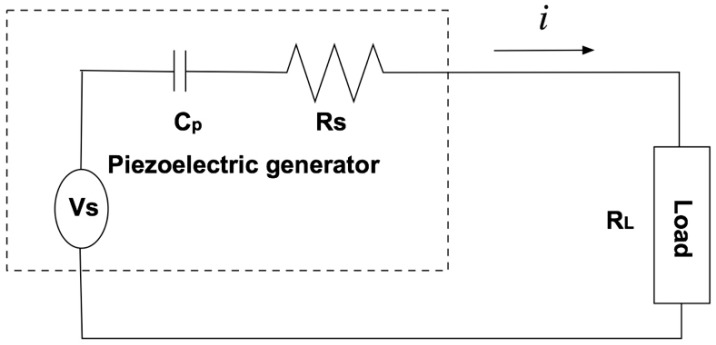
Equivalent circuit for the piezoelectric harvester connected with a pure resistive load. The dash lined rectangle represents the piezoelectric harvester.

When an external resistive load ($R_{L}$) is connected to the piezoelectric harvester, as shown in Figure 2, the voltage across the resistive load is maximum when the load resistance ($R_{L}$) is matched to the source impedance ($Z_{p}$). If the piezoelectric material is an ideal capacitor and the dielectric loss of the piezoelectric structure is infinitely small, the voltage across the resistive load can be obtained as follows:

(14) $$V_{\text{L}}=V_{\text{OC}}\left|\frac{R_{\text{L}}}{Z_{p}+R_{\text{L}}}\right|$$

where $Z_{p}\approx {\left(\omega C_{p}\right)}^{-1}$, and the maximum electrical voltage to a resistive load is half of the open circuit voltage, (*i.e.*, $V_{L,max}\;=\;V_{OC}/2$). Under dynamic conditions (simple sinusoid), the RMS power delivered to the resistive load is given by:

(15) $$P_{\text{rms}}=\frac{V_{\text{L}}^{2}}{2R_{\text{L}}}$$

The RMS voltage for a time dependent signal can be calculated directly from the following equation:

(16) $$V_{rms}=\sqrt{\frac{1}{T}\int_{0}^{T}V{\left(t\right)}^{2}\;dt}$$

## 3. Flow Loop Experimental Setup

**Figure 3:**
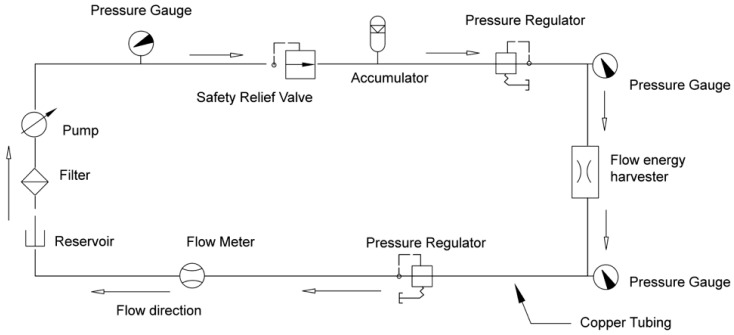
Schematic of the flow loop that is currently being used to measure the performance of the harvesters (reprinted with permission from [18]; copyright 2014 SPIE Publications).

A schematic of the flow loop used in experiments is shown in Figure 3. The loop contains a motor/pump and a reservoir, and has digital pressure gauges (G1 Pressure transducers manufactured by ASHCROFT, Stratford, CT, USA) on the inlet and outlet of the flow energy harvester test section. For the measurement of flow rate, a digital flow meter (Stainless Steel Flowmeter with 4–20 mA module, GPI, Wichita, KS, USA) is used and is located at the reservoir inlet. A safety relief valve is positioned between the pump and the accumulator. Accumulator is used to reduce vibration and noise from the pump. Pressure regulators are placed before and after the flow energy harvester. The pump speed and flow rate are adjusted dictating the harvester test section inlet and outlet pressures. A filter between the reservoir and the pump is used to catch particles in the flow. The tubing is made of copper and the joints are connected with AN fittings. The back-pressure on the flow energy harvester test section can be maintained above a critical level to suppress cavitation via a needle valve.

Once the test section is fitted accordingly, the pump motor rotating speed was set and the flow rate, inlet, and outlet pressures were recorded. For power output measurements, the load resistance across the electrical output of the harvester and corresponding voltage V were measured using an oscilloscope (TDS 2024B, Beaverton, OR, USA). The waveform of V in time was downloaded to the computer and the instantaneous power was time averaged to find the average maximum recoverable power generated.

**Figure 4:**
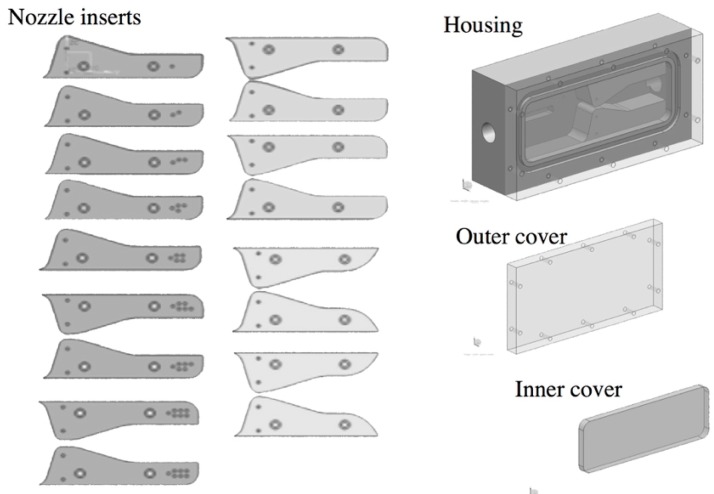
CAD models of the mechanical cantilever housing and a variety of spline nozzles for testing the fluid structure interaction.

Nozzle-diffusers, the housing, and mechanical cantilever designs for one set of tests are shown in Figure 4. Different spline-shaped nozzle-diffuser configurations were designed with varying throat sizes. In order to accommodate the maximum flow loop pressure (1.7 MPa), the harvester housings were made of aluminum and the flow profiles (nozzle-diffuser inserts) were mounted with dowel pins and screws. An o-ring was inserted into a groove at the front face of the housing, and a Plexiglass cover was then screwed-in against the o-ring to seal the surface.

## 4. Bimorph Harvester

A piezoelectric bimorph connected in parallel (QP21B produced by Mide Technology Corporation, Medford, MA, USA [26]) was used to demonstrate power generation in our first flow energy harvester design. This bimorph consists of two thin 0.008 inch (0.2032 mm) of PZT5A type piezoelectric elements (3195HD) that are 1.33 inch (33.782 mm) long and 0.56 inch (14.224 mm) wide. They are covered with thin 0.001 inch (0.0254 mm) polyimide layers to protect and electrically isolate the electrodes.

**Figure 5:**
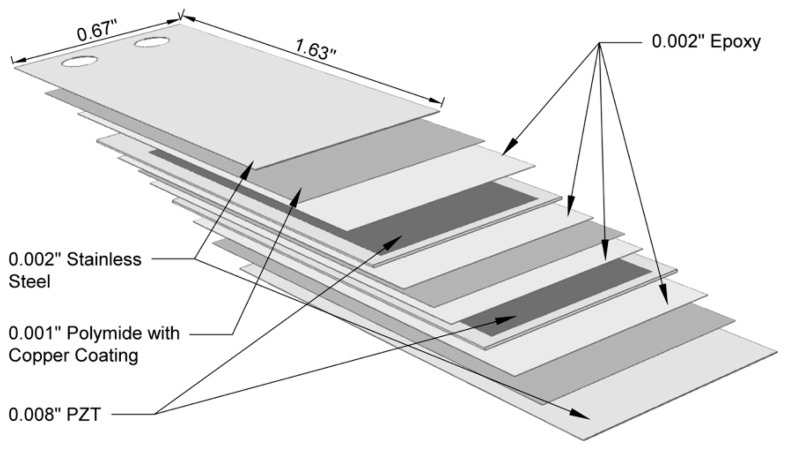
An exploded view of the armored QuickPac (QP21B) actuator.

**Figure 6:**
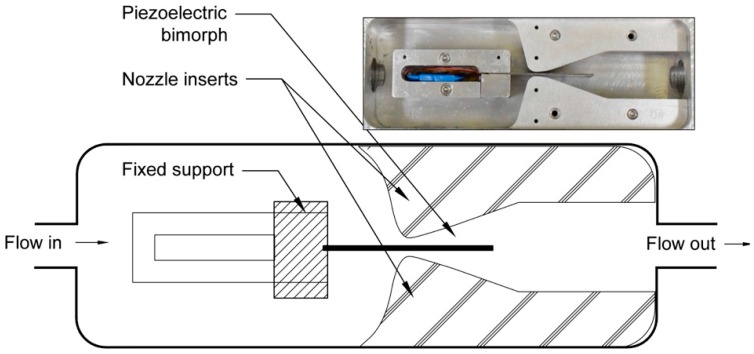
Photograph and schematic representation of a flow energy harvester based on piezoelectric bimorph and the spline nozzle. Flow is left to right and the nozzle profiles can be adjusted by replacing the nozzle inserts.

A 0.002 inch (0.0508 mm) stainless steel shim was added to either side of the commercially available QP21B bimorph actuator in order to increase its fatigue life and erosion resistance (armored QP21B). An exploded view of the armored QP21B actuator is shown in Figure 5, where the total length and width are 1.63 inch (42.418 mm) and 0.67 inch (17.018 mm), respectively. The total thickness is 0.031 inch (0.7874 mm). A photograph and schematic illustration of our typical bimorph flow energy harvester are shown in Figure 6.

**Figure 7:**
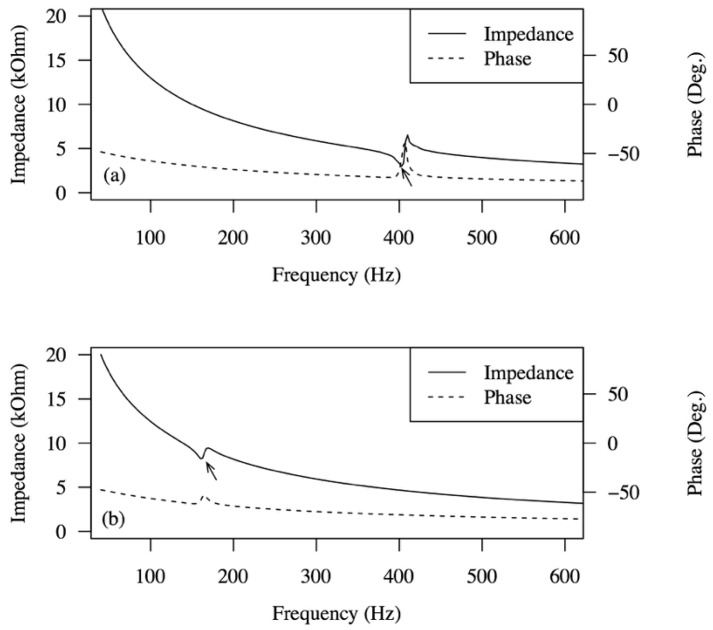
Electrical impedance and phase spectra of armored QP21B as a function of frequency measured in air (**a**) and in water (**b**). Arrow indicates the location of resonant frequency.

Figure 7 shows the electrical impedance and phase spectra of armored QP21B in air and in water as a function of frequency, measured using an HP4294A impedance/gain-phase analyzer (Hewlett-Packard, Palo Alto, CA, USA). This impedance analysis determines the target resonant frequency to be excited by the flow as well as the optimum resistance of the load resistor. Note that the first resonant frequency of the armored QP21B is located at around 400 Hz in air, but decreases to around 140 Hz in water. Its optimal electrical resistance at 140 Hz in water is between 7 and 10 kOhm.

**Figure 8:**
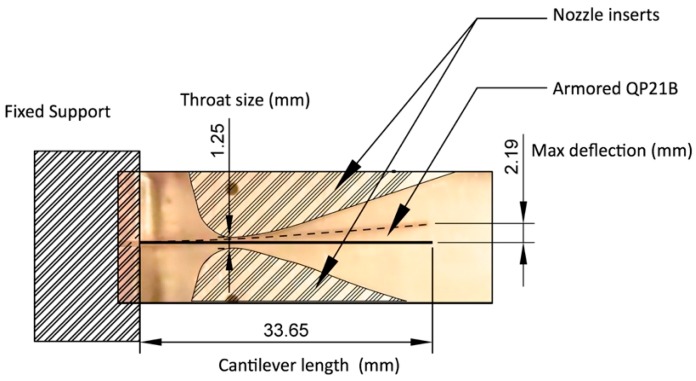
Snapshots and deflection analysis of the motion of armored (2 mil steel) QuickPac (QP21B) inside flow harvester body under a flow rate of 15 L/min using high speed camera (Frame rate = 1200 fps). The snapshot shows the frame when the deflection of the QP21B is the maximum.

For power output measurements of the bimorph, the harvester system is placed in the flow loop described. An example snapshot showing the deflection of piezoelectric bimorph at its maximum can be found in Figure 8. This frame was taken at a flow rate of 15 L/min with a nozzle throat size of 1.25 mm, using a high speed (1200 frame per second) camera (Nikon 1 J4, Chiyoda, Tokyo, Tokyo, Japan). As shown, the max deflection of this bimorph is found to be on the order of 2 mm.

**Figure 9:**
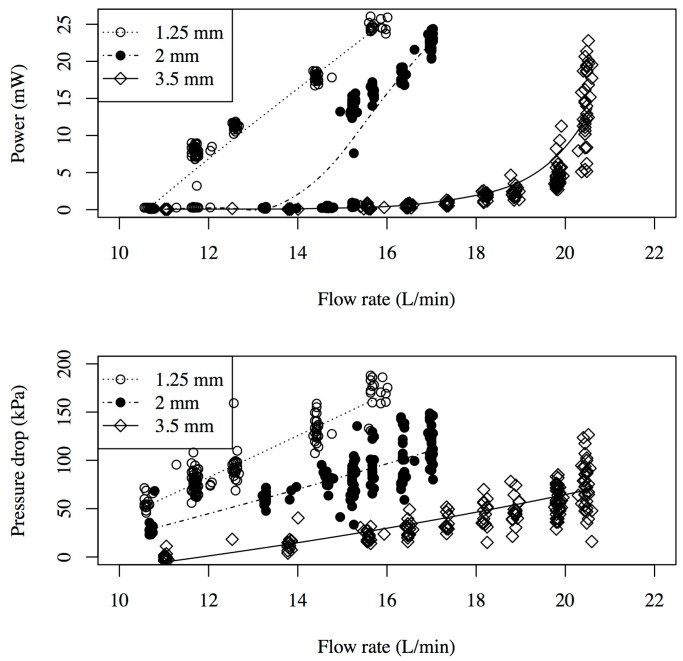
The power and pressure drop as a function of the flow rate for various nozzles with gap sizes ranging from 1.25 mm to 3.5 mm. The lines are determined from the least squares regression analysis.

The measured power from the armored QP21B for different nozzle throat sizes is shown in Figure 9. Note that the output power with all tested nozzle-diffuser inserts showed the same maximum of 25 mW albeit at different flow rates (ranging from 10 to 20 L/min). Notice that the data clearly shows a critical flow rate exists at which power generation increases rapidly (“turns on”), then appears to further increase linearly with increasing flow rate. The larger the throat size the larger this critical flow rate. Another trend in the data is an increase in throat size leads to a decrease in the average pressure drop across the test section; for example, at large nozzle throat size of 3.5 mm one could only generate non-negligible amounts of power near the maximum flow rate of 20 L/min. Yet the pressure drop is much smaller than the subsequent runs with smaller nozzle throat sizes (35–70 kPa for the larger throat).

**Figure 10:**
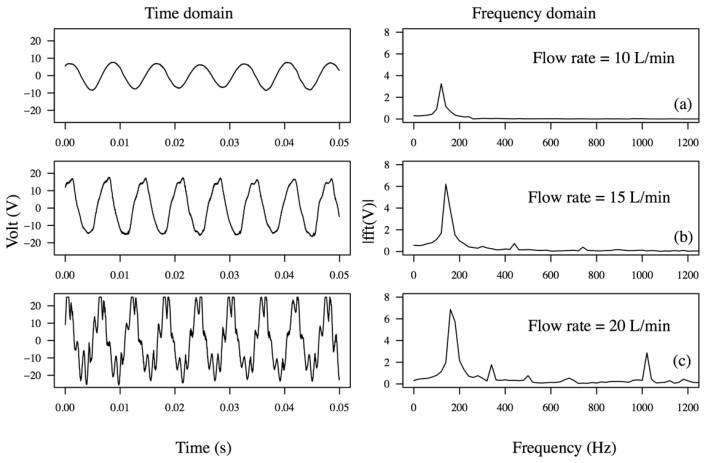
Voltage waveforms of armored QP21B harvester in the time domain and frequency domain depending on the flow rate when connected with a load of 10 kOhm. Corresponding power outputs at 10 L/min, 15 L/min and 20 L/min are 2.36 mW, 13 mW and 22 mW, respectively.

Figure 10 shows the armored QP21B voltage waveforms for multiple flow rates and a nozzle throat size of 1.25 mm. 

**Figure 11:**
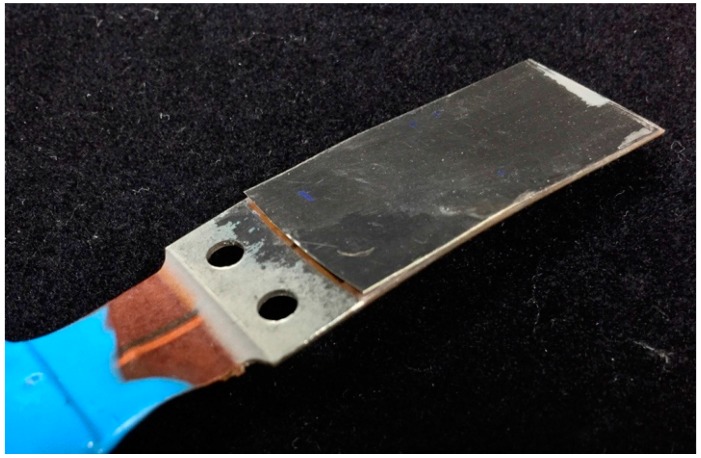
The photograph of armored QP21B after 9 hour life test.

The frequency-domain signal of the voltage waveforms were also obtained by taking their respective fast Fourier transform (FFT). Notice that the dominant frequency at 15 L/min corresponds to the first mode, located between 140 and 150 Hz; however, as the flow rate is increased, the voltage signal shows multifrequency response peaks, exhibiting first (140 Hz) and second (1 kHz) resonance mode vibrations. Although not shown in Figure 9, by further increasing the flow rate to 20 L/min power levels up to 35 mW could be generated, yet the life of the device, as expected, was found to be short. Testing at reduced flow rates, under 10 L/min, produced 5–6 mW and was shown to run for 9 h without failure (3,888,000 cycles); however after 9 hours a crack in the metal shim developed where the actuator was clamped. The photograph of the armored QP21B after the 9 h life test, shown in Figure 11, suggests that the cause of the failure was that the actuator was being driven past its metal shim fatigue limits.

The crack location suggests that the rigid fixture and the right angle joint clamping the bimorph may have been responsible for stress concentrations that caused the fatigue failures observed. In order to investigate the effect of mounting on the fatigue of the piezoelectric cantilever, a stress analysis was performed using the finite element method. Note that the fatigue strengths of the strainless steel and piezoelectric material in the bimorph actuators are around 210 MPa and 50 MPa, respectively, below which fatigue failure is not expected [27,28]. Hence, the fatigue failure of the bimorph harvester can be eliminated with a design that ensures cyclic stresses are sufficiently lower than these limits.

**Figure 12:**
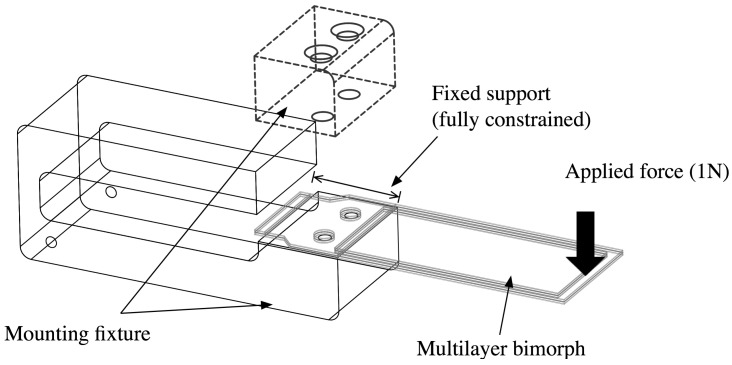
Geometry of armored QP21B model with mounting fixture. Boundary conditions for the model is shown in figure.

To develop the finite element model of the multilayered, armored QP21B, the commercial finite element software package ABAQUS was used. A geometric model of the armored QP21B was created using Siemens PLM Software NX software, and then imported into ABAQUS for the stress analysis. Figure 12 shows the geometry of the model with applied the load and fixed boundary conditions. The FEA model includes epoxy layers, Espanex polyimide layers, stainless steel shims and the piezoelectric layers, whose properties are summarized in Table 1 and Table 2. Hexahedral (brick) elements with reduced integration are used for both piezoelectric elements (C3D8E) and non-piezoelectric layers (C3D8R). All the layers are meshed with an element size of 0.4 mm in length and width, and four elements along the thickness direction. A finer mesh, 0.2 mm in element size, was used in the region where the actuator was mechanically clamped in order to improve the accuracy of localized stress concentrations. 

**Table 1:** Material parameters of non-piezoelectric layer.

Property	Epoxy	Polyimide	Stainless Steel
Density (kg/m^3^)	0.73	1.42	7800
Young’s Modulus (GPa)	3.5	2.5	200
Poisson’s ratio	0.35	0.34	0.3

**Table 2:** Piezoelectric properties (CTS-3195HD) from Mide Technology Corporation.

Property	Symbol	Value
Density (kg/m^3^)	ρ	7800
Relative permittivity	$\varepsilon_{11}^{s}/\varepsilon_{0}\;,\;\varepsilon_{33}^{s}/\varepsilon_{0}$	916, 830
Compliance (pm^2^/N)	${\text{s}}_{11}^{D}$, ${\text{s}}_{12}^{D}$, ${\text{s}}_{13}^{D}$, ${\text{s}}_{33}^{D}$, ${\text{s}}_{44}^{D}$	14.4, −4.24, −2.98, 9.43, 25.2
Piezoelectric charge constants (pC/N)	d_33_, d_31_, d_15_	390, −190, 585

**Figure 13:**
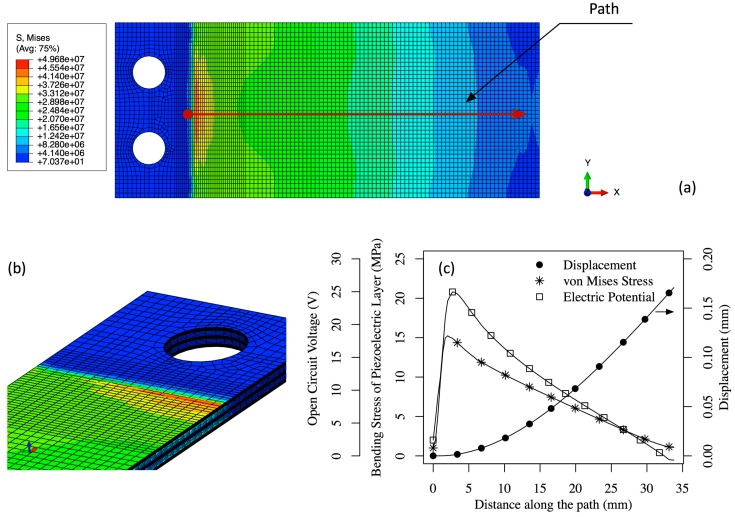
The von Mises stress distribution (MPa) of top stainless steel plate (**a**) and cross section (**b**) for the armored (2 mil steel) QuickPac (QP21B) at a tip force of 1N; (**c**) shows bending stress, tip displacement and open circuit voltage of the top piezoelectric layer of QP21B along the path shown in (**a**).

Figure 13a,b show the static analysis results exposing the stress concentrations at the clamped joint of the armored QP21B. A load of 1 N is applied at the tip of the beam and causes a tip deflection of around 0.18 mm. The stainless steel layer shows the highest stress level (50 MPa), in part due to the higher Young’s modulus of stainless steel compared to other layers. The piezoelectric layer exhibits a maximum von Mises stress level of around 15 MPa. The electric potential (open circuit voltage), maximum bending stress, and tip displacement of the piezoelectric layer as a function of position along the actuator length are shown in Figure 13c, with an open circuit voltage of 20 V at a tip deflection of 0.18 mm. The electrical RMS power output can be estimated at 5 mW from Equation (15), assuming that the bimorph is vibrating at 150 Hz, that the matched electrical impedance is 10 kOhm, $R\approx {\left(\omega C_{p}\right)}^{-1}$, and $V_{L}=10\;V$. The stiffness can be estimated from these results using its definition, $k=\frac{F}{\delta }$, and the effective Young’s modulus of the armored QP21B struture can be calculated according to Equation (6). The former and latter values are 5.55 N/mm and 205 GPa, respectively.

**Figure 14:**
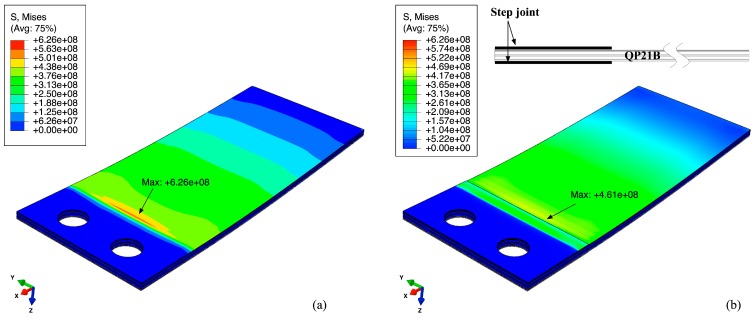
The von Mises stress distribution of armored QP21B without a stepped joint (**a**) compared with that of armored QP21B with a stepped joint (**b**). The thickness of a stepped joint is the same as that of protective stainless steel layer (t = 0.0508 mm).

Under an unsteady fluid flow driving force, the armored QP21B tip deflection reaches an approximate maximum of 2 mm (see Figure 8), meaning that the bending stresses on the structure are about 10 times higher than those predicted in the previous analysis. For example, the stress on the stainless steel layer would be on the order of 500 MPa, which is above the fatique limit of the material. A variety of methods to reduce the stress concentration has been investigated, including adding a fillet or a glue bond line that extends along the mounting edge line of the bimorph. The stepped joint mounting design is found to be simple and effective in reducing these stress concentrations without affecting the bending stiffness of the structure. Figure 14 shows the stress analysis results of armored QP21B with a stepped joint (0.0508 mm in thick) under a 2 mm tip displacement. For comparison, the stress analysis result of armored QP21B without a stepped joint under a 2 mm tip displacement is also shown in the figure. Note that as expected, extending the mount and stepping it can have a 26% reduction in the stress field at the mounting line.

An alternate means of calculating power output and a first order approximation of the stress profile along the length of different layers of the bimorph actuator used video data that mapped the beam shape. Although the method needs refinement, this section aims at discussing its details and preliminary results as compared to the finite element simulations and experimentally measured power output. The analysis considers the cantilever as a forced Euler-Bernoulli beam with clamped- and free-end boundary conditions. This implies that bending stresses are dominant over shear stresses, and that small strain and small beam displacement relative to the beam length L exist. The shape of the beam was approximated by an eigenfunction expansion that satisfies the above boundary conditions for the eigenvalue problem of the biharmonic operator [29]:

(17) $$\phi_{n}(x)=\cosh(\beta_{n}x)-\cos(\beta_{n}x)+a_{1}(\sin(\beta_{n}x)-\sinh(\beta_{n}x))$$

where the wave number $\beta_{n}$ satisfies the constraint:

(18) $$\cos(\beta_{n}L)\cosh(\beta_{n}L)=-1$$

and the constants are:

(19) $$a_{1}=\frac{\left(\cos(\beta_{n}L)+\cosh(\beta_{n}L)\right)}{\left(\sin(\beta_{n}L)+\sinh(\beta_{n}L)\right)}$$

with x as the coordinate along the beam length. The displacement δ is:

(20) $$\delta (x,t)=\sum_{n=1}^{\infty }\xi_{n}(t)\phi_{n}(t)$$

where $\xi_{n}\left(t\right)$ is a periodic function of time.

**Figure 15:**
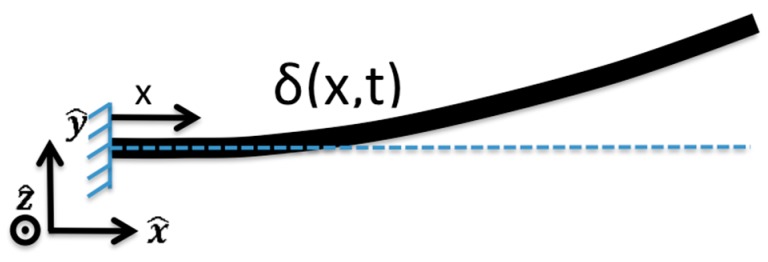
Illustration of deformed beam represented by δ(x,t) with coordinate system (reprinted with permission from [18]; copyright 2014 SPIE Publications.)

Figure 15 shows the beam deflection δ as a function of x and time t.

Camera data taken as slow motion video (Nikon 1 J4, 1200 frame per second) was processed frame by frame and the edges of the vibrating cantilever were mapped in the x-y plane, shown in Figure 15, using the Canny edge filter implementation in MATLAB [30]. The filter parameters were chosen as necessary to consistently capture the edges near the same location (±1 pixel) for each video file. The actuator experiments chosen for the initial processing consisted of vibration almost entirely in actuator’s fundamental mode. Hence, only the n=1 solution to Equation (17) is considered for the cases shown. At each frame, the x-y actuator edge data is least squares fitted to Equation (16), yielding a constant coefficient for $\xi_{1}\left(t_{i}\right)$, where index i is the frame number. $\xi_{1}\left(t\right)$ is a sinusoid fit to the time series $\xi_{1}\left(t_{i}\right)$ with the appropriate frequency and phase parameters. 

**Figure 16:**
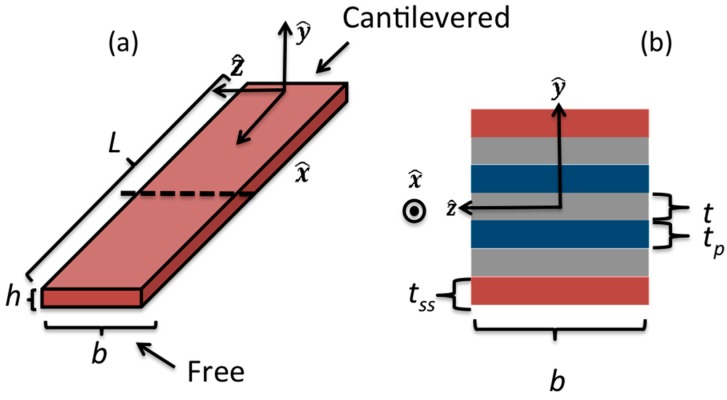
Illustration of (**a**) bimorph and (**b**) cross-section. Blue represents the piezoelectric material layer, grey the polyimide coating, and red the 301 stainless steel layer for the geometry of the armored QP21B actuator (reprinted with permission from [18]; copyright 2014 SPIE Publications).

As shown by Sodano *et al.* [31], the power output can be calculated from δ(x,t). The piezoelectric constituent equations applied to the bimorph actuator geometry yields a first order ODE for the charge q(t) as:

(21) $$R\dot{q}(t)-C_{p}^{-1}\Theta_{i}\xi_{i}(t)+C_{p}^{-1}q(t)=0$$

where R is the circuit resistance and the dot represents the derivative in time. The capacitance is:

(22) $$C_{p}={∭}_{V_{p}}\psi^{2}(y)\varepsilon dV$$

with $V_{p}$ as the bounds to the piezoelectric volume. The electromechanical coupling constant is:

(23) $$\Theta_{1}=-{∭}_{V_{p}}ye\phi_{1}^{\prime \prime }(x)\psi (y)dV$$

where e is the piezoelectric coupling coefficient, y is the coordinate variable defined from the neutral plane of the beam, and the function:

(24) $$\psi (y)=\{\begin{array}{cc} -\frac{1}{t_{p}}, & \frac{t}{2}<y<\frac{t}{2}+t_{p}\\ 0, & -\frac{t}{2}<y<\frac{t}{2}\\ \frac{1}{t_{p}}, & -\frac{t}{2}-t_{p}<y<-\frac{t}{2} \end{array}$$

for y values shown in Figure 16b.

Variation of parameters yields the steady state solution to Equation (21):

(25) $$q_{ss}=\int_{0}^{t}\exp[{\left(RC_{p}\right)}^{-1}(t-\tau )]{\left(RC_{p}\right)}^{-1}\Theta_{1}\xi_{1}(\tau )d\tau$$

and the instantaneous power output P:

(26) $$P(t)={\dot{q}}_{ss}^{2}R$$

**Figure 17:**
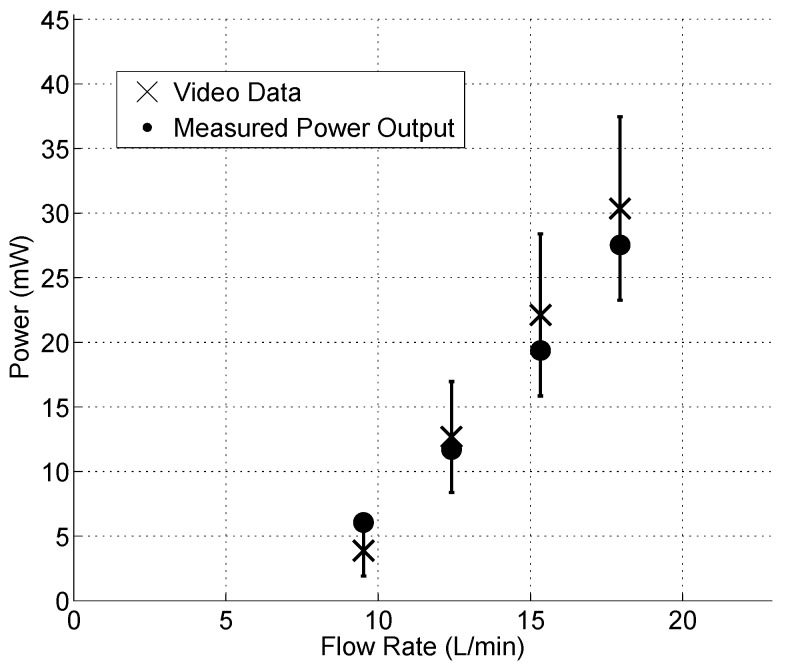
Video processed power data (x) and oscilloscope measurement (dots) (reprinted with permission from [18]; copyright 2014 SPIE Publications).

Video for the armored QP21B was analyzed for the experiments with a nozzle throat size of 1.25 mm and flow rates of 9.5 L/min, 12.4 L/min, 15.3 L/min, and 18 L/min. The power generated is shown in Figure 17. The piezoelectric coupling coefficient is e=1.5052E−08 m/V and resistance is R=10 kOhms. The figure shows the results of the analysis, plotting the predicted power output with an error bound of ±1 pixel and the time average of RMS oscilloscope measured voltage and derived power (error ranges from ±1.4 mW at the lowest flow rate to ±7.1 mW at the highest). 

**Figure 18:**
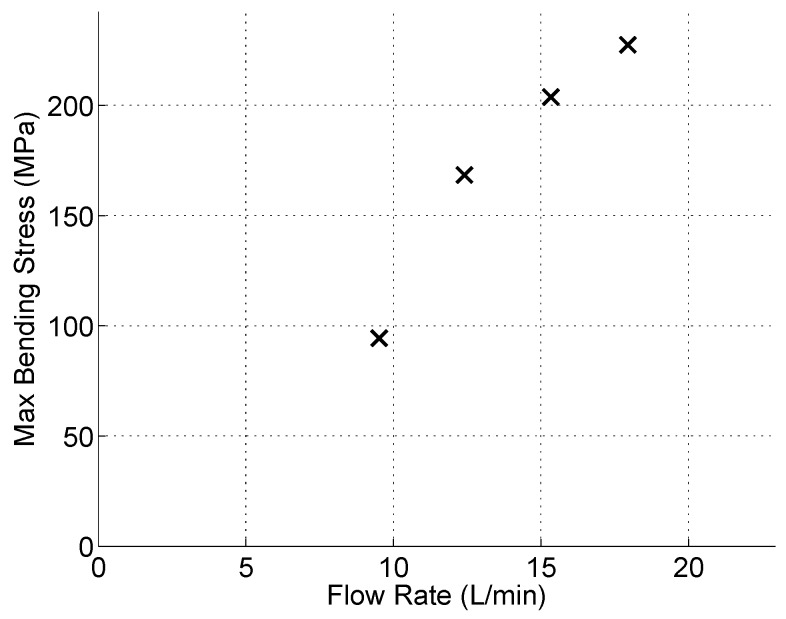
Maximum bending stress on the stainless steel layer of bimorph actuator.

**Figure 19:**
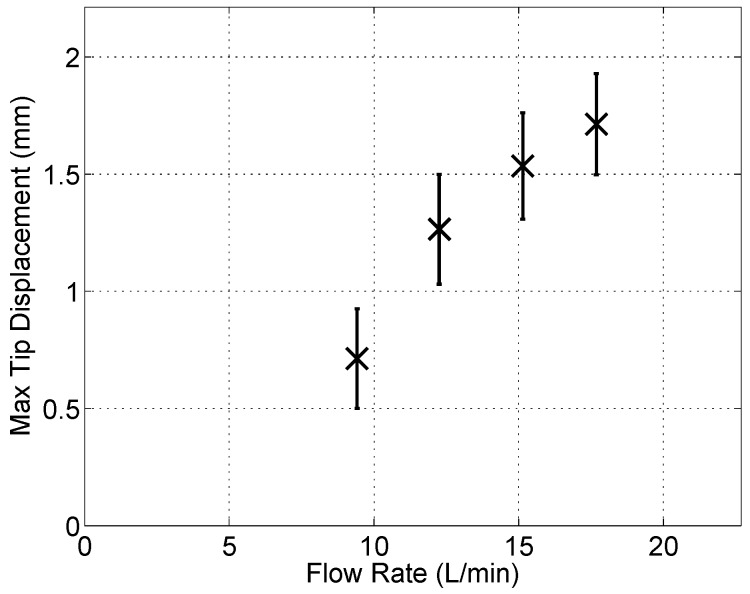
Maximum measured tip displacements from video data.

Although the model is limited to assumptions of the Euler-Bernoulli beam and the boundary conditions mentioned, it seems to do a reasonable job at predicting the measured RMS power. Consequently, stresses in the stainless steel can be estimated from the fitted eigenfunction as:

(27) $$\sigma_{ss}=\frac{My}{I}$$

where is the M is the bending moment:

(28) $$M=EI\frac{\partial^{2}\delta }{\partial x^{2}}$$

and I is the area moment of inertia for each section shown in Figure 16. For the flow rates tested, Figure 18 shows the maximum bending stresses in the stainless steel section. The corresponding tip displacements from the processed video data are shown in Figure 19. From the FEA results shown in Figure 14, the stress level magnitudes of contours outside of the stress concentration zone are within the ballpark of the surface stresses calculated using the video data. For example, with a tip displacement of ~1.68 mm, the maximum surface bending stress of the stainless-steel element is ~238 MPa.

## 5. Double Flextensional-Cantilever Harvester

**Figure 20:**
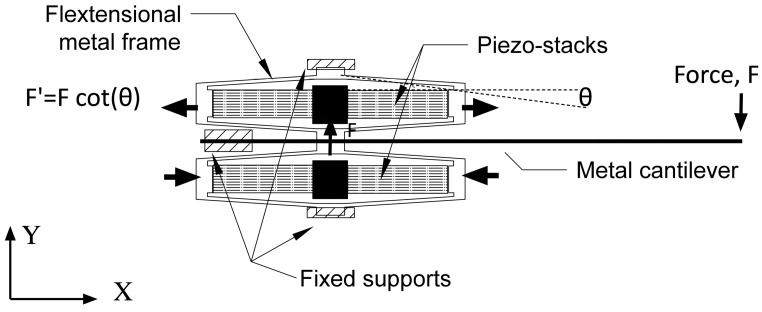
Schematic representation of double flextensional harvesters, which are mechanically connected to a metal cantilever. The arrows indicate the displacement directions. Boundary conditions for the model is shown in figure.

A second promising prototype is the Double Flextensional Cantilever Harvester (Double- FCH). A schematic representation of the Double- FCH is shown in Figure 20, where the metal cantilever is mounted and coupled between two flextensional actuators. The principle of operation is the flow-induced vibration onto the non-piezoelectric cantilever transfers forces into the flextensional frame along the y axis (minor axis). The frame, in turn, amplifies the forces along its x axis (major axis). The force amplification is related to the ratio of the long axis length to the short axis height, which can be approximated as shown in Figure 20, (*i.e.*, $F_{x}=F_{y}\cot\theta$).

Commercially available APA 400M flextensional actuators (Cedrat Technologies S.A., Meylan, France) were selected due to their resonant frequency of ~350 Hz under a blocked-free boundary condition. This resonant frequency can be tuned further with an added mass at either support point of the flextensional actuators. In order to use these flextensional actuators in water, the piezoelectric stacks were replaced with water resistant stacks purchased from American Piezoelectric Ceramic (Mackeyville, PA, USA) APC − 30 × 45 − 1130 Pst150/5 × 5/20. The stack has the cross section 5 mm × 5 mm and a length of 18 mm.

Finite element analysis of the Double-FCH was performed using ABAQUS in order to predict its performance. An assumption for the model consisted of the stack as an isotropic material, with effective Young’s modulus and dielectric permittivity derived based on the measured and reported dimensions, stiffnesses, piezoelectric strain coefficients and capacitances of stacks [32] (shown in Table 3). Piezoelectric materials were meshed with a global element size of 0.5 mm, generating 14400 hexahedral linear elements (C3D8E), while non-piezoelectric materials were meshed with total 53064 hexahedral linear elements, which have a global element size of 0.5 mm with finer elements in the thickness of flextensional frame and cantilever beam.

**Table 3:** Material parameters of piezoelectric multlayer stack (APC − 30 × 45 − 1130 Pst150/5 × 5/20).

Materials	l/w/h	k	C	ε_33_^T*^	d_33_*	Y_l_
	(mm)	(N/um)	(µF)	(F/m)	(m/V)	(Gpa)
Pst150/5 × 5/20	5/5/18	60	1.63	0.00117	8.41E−08	43

**Figure 21:**
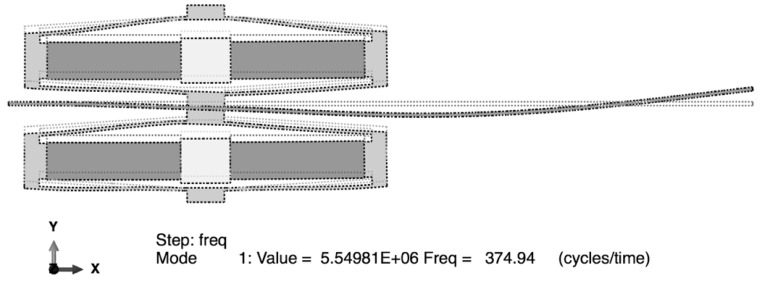
Deformed shape of the Double-Flextensional-Cantilever Harvester with superimposed undeformed shape (dahsed line) under short circuit condition. The resonant frequency is 374.9 Hz.

A modal analysis was performed in order to identify the mode shapes, the resonant frequencies and electromechanical coupling of the Double-FCH structure. Figure 21 illustrates the deformed shapes of the double-FCH at resonance (374.9 Hz) in a short circuit condition. The undeformed shape is superimposed on the deformed shape. An open circuit modal analysis was also performed by removing the voltage boundary condition, and its first natural frequency in resonance found to be 385.5 Hz. Note that the resonance frequency $f_{r}$ represents the mechanical resonance vibrating under short-circuit conditions, while the anti-resonance frequency $f_{a}$ represents the mechanical resonance vibrating under an open-circuit condition, indicating that *f_r_* and *f_a_* are 374.9 Hz and 385.5 Hz, respectively. The effective electromechanical coupling factor can be calculated using Equation (29), and found to be 0.23. Note that higher electromechanical coupling factor implies better mechanical coupling between flextensional actuators and cantilever beam, allowing for effective vibration transfer from the cantilever beam to the flextensional actuators, and to piezoelectric stacks:

(29) $$k_{eff}=\sqrt{1-{\left(\frac{f_{r}}{f_{a}}\right)}^{2}}$$

**Figure 22:**
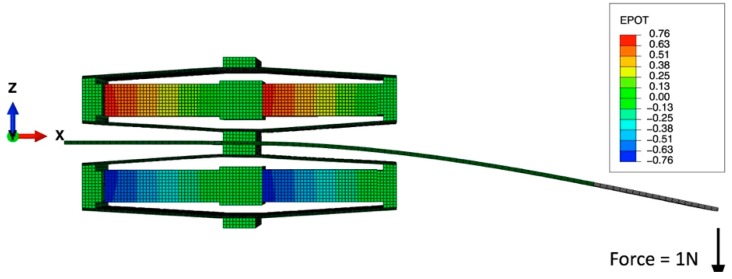
Static analysis of double- flextensional-cantilever harvester (FCH), showing open circuit voltage (EPOT) under a load of 1 N.

Figure 22 shows the static analysis results, exhibiting the open circuit voltage from the piezoelectric stacks when a load of 1 N is applied at the free end of the beam. The reaction forces at the piezoelectric stack are found to be 0.8 N along the y direction, but are amplified to 10 N along the x direction due to the frame lever arm magnification of the applied force. Note that the open circuit voltage can be analytically calculated using the Equation (10), where $d_{33}$, $C_{p}$ and $F_{x}$ are 0.0841 µm/V, 1.63 µF and 10 N, respectively, yielding around 0.7 V. An estimate of power output can be made assuming that the structure is vibrating at its natural resonant frequency of 374 Hz and has a matched resistance $R_{L}$) of 235 Ohm based on $R_{L}={\left(\omega C_{p}\right)}^{-1}$. The generated instantaneous power output per a stack would be around 0.2 mW, and since the FCH is composed of 4 stacks, the FCH yields a total of around 0.8 mW. This is a factor of 5–6 smaller than when compared to that of the armored QP21B harvester. The lower power with respect to applied force is due to the stack’s higher stiffness (60 N/um) as compared to the armored QP21B (5.5 N/um). Note, however, that this design can survive higher stress levels than the bimorph actuator and thus produce higher power when an appropriate flow passage and cantilever design provides a frequency-matched, high amplitude (~10 N) fluid-structure forcing function. Another method to further increase output power is to use a lower stack stiffness. Since capacitance is inversely proportional to the cross-sectional area of the stack, the use of a stack with smaller cross-sectional area allows for higher output power under the same boundary conditions assuming that the applied force and piezoelectric charge coefficient are kept constant (see Equation (30)):

(30) $$P=\frac{\omega d_{33}^{2}}{2C_{p}}F^{2}$$

**Figure 23:**
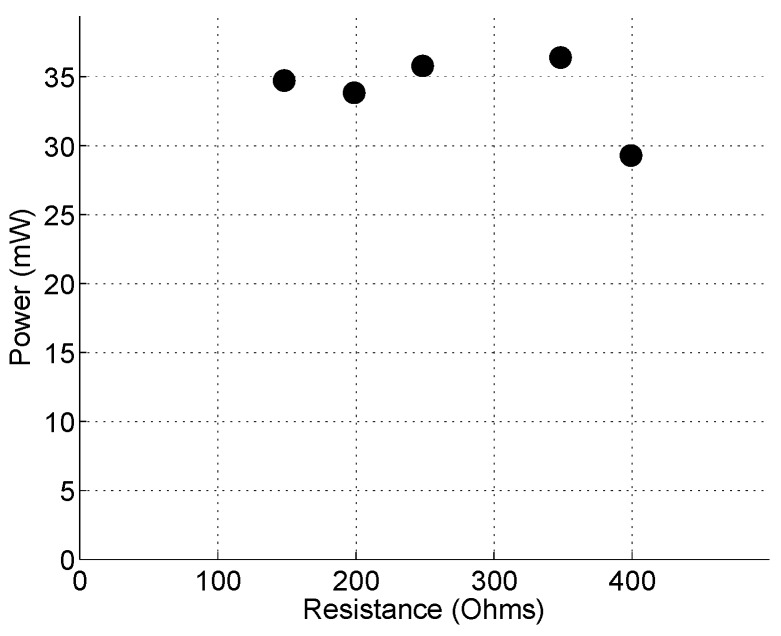
The power produced from one flextensional actuator of double-Flextensional Cantilever Harvester (FCH) driven by compressed air as a function of the load resistance. The power level at each resistance roughly corresponds to the maximum inlet pressure of 410 kPa.

The Double-FCH performance was determined experimentally in both air and water. The generated output voltages were investigated as a function of the load resistance at the various pressure levels in air first. Figure 23 shows the corresponding power output from a single flextensional actuator as a function of the resistance. The power shows a flat peak of about 35 mW at about 200–350 Ohms at the maximum inlet pressure of 410 kPa, and was decreased above 350 Ohm. A flat peak in the range of 200–350 Ohm, rather than a sharp peak at a matched resistance (~235 Ohm), is believed to be due to combined resonance effects from both cantilever and flextensional actuators. This induces multi-frequency harmonic excitations on piezoelectric materials, resulting in a flat peak in the range of 200–350 Ohm. Note that since this power is from a single flextensional actuator equipped with two stacks, the flow energy harvesting device is technically capable of generating 70 mW (four stacks per flow energy harvester).

**Figure 24:**
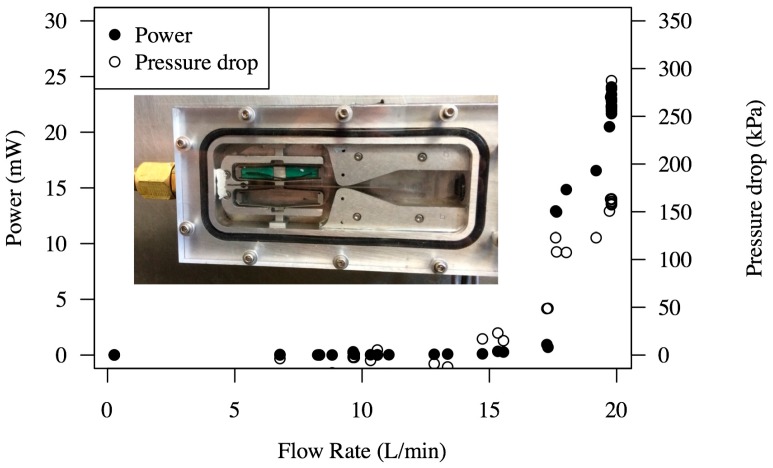
Power and pressure drop of double-Flextensional Cantilever Harvester (FCH) as a function of the flow rate (L/min). The measurement was from a single flextensional actuator. The inset shows a photograph of tested FCH.

**Figure 25:**
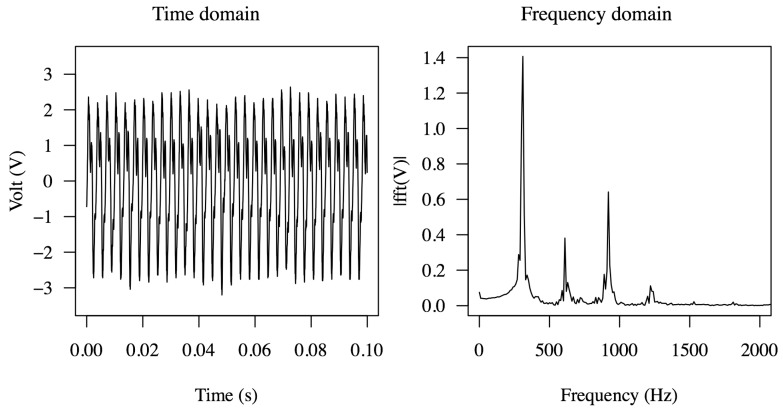
Voltage waveform in the time domain and frequency domain across of 100 Ohm resistor obtained from one flextensional actuator of double-Flextensional Cantilever Harvester (FCH) tested in water. RMS voltage of double-FCH is 1.5 V.

The Double-FCH was then tested in the flow loop system described. The measured power and pressure drop as a function of the flow rate are shown in Figure 24. The maximum power corresponds to about 25 mW across a 100 Ohm resistor at a flow rate of 20 L/min and a pressure drop of 165 kPa. It should be noted that to achieve this power level, the cantilever was shortened to 90 mm from the original 100 mm tested in air. The voltage waveform and corresponding frequency content across a 100 Ohm resistor at a flow rate of 20 L/min are shown in Figure 25, where the dominant frequency excited for this harvester is found to be about 305 Hz with ~1.5 of RMS voltage. 

## 6. Conclusions and Future Work

Two designs of piezoelectric transducers were investigated in this study. Both the bimorph- and flextensional-based flow energy harvesters rely on fluid motion coupled to structural vibration via a cantilever placed in a converging-diverging flow channel. The bimorph is the cantilever itself, while the flextensional clamps a non-piezoelectric cantilever that provides the forces it converts into electricity. The two designs experimentally generated power at a level of 20 mW and above, with the bimorph type harvester specifically prone to fatigue failure caused by stress concentrations at its mounting point. A stepped joint mounting design was shown via FEA to ameliorate this issue, with a reduction of 26% in stress concentration without a reduction in power output.

The flextensional actuator based harvester was found to be a viable alternative to the bimorph, with a power generation of ~20 mW from a single flextensional actuator. Greater robustness (unexposed piezoelectric to flow) and more design flexibility (cantilever can be designed independently of piezoelectric fatigue limits) are advantages the flextensional type harvester (FCH) provides. These results have prompted further investigation into different designs using this type of actuator. For example, the ability to tune the resonance frequency of the actuator by adding mass to the flextensional bodies may allow another design parameter that can be optimized to maximize power output. Further research is currently underway in order to increase fluid-structure coupling efficiency and further ruggedize harvesters.
